# Pharmacological Investigations of the Dissociative ‘Legal Highs’ Diphenidine, Methoxphenidine and Analogues

**DOI:** 10.1371/journal.pone.0157021

**Published:** 2016-06-17

**Authors:** Jason Wallach, Heather Kang, Tristan Colestock, Hamilton Morris, Zuner A. Bortolotto, Graham L. Collingridge, David Lodge, Adam L. Halberstadt, Simon D. Brandt, Adeboye Adejare

**Affiliations:** 1 Pharmaceutical Sciences, Philadelphia College of Pharmacy, University of the Sciences, Philadelphia Pennsylvania, United States of America; 2 Centre for Synaptic Plasticity, School of Clinical Sciences, University of Bristol, Bristol, United Kingdom; 3 Centre for Synaptic Plasticity, School of Physiology, Pharmacology and Neuroscience, University of Bristol, Bristol, United Kingdom; 4 Department of Physiology, Faculty of Medicine, Toronto, Ontario, Canada; 5 Lunenfeld-Tanenbaum Research Institute, Mount Sinai Hospital, Toronto, Ontario, Canada; 6 Department of Psychiatry, University of California San Diego, La Jolla, California, United States Of America; 7 School of Pharmacy and Biomolecular Sciences, Liverpool John Moores University, Byrom Street, Liverpool, United Kingdom; Hudson Institute, AUSTRALIA

## Abstract

1,2-Diarylethylamines including lanicemine, lefetamine, and remacemide have clinical relevance in a range of therapeutic areas including pain management, epilepsy, neurodegenerative disease and depression. More recently 1,2-diarylethylamines have been sold as ‘legal highs’ in a number of different forms including powders and tablets. These compounds are sold to circumvent governmental legislation regulating psychoactive drugs. Examples include the opioid MT-45 and the dissociative agents diphenidine (DPH) and 2-methoxy-diphenidine (2-MXP). A number of fatal and non-fatal overdoses have been linked to abuse of these compounds. As with many ‘legal highs’, little is known about their pharmacology. To obtain a better understanding, the effects of DPH, 2-MXP and its 3- and 4-MeO- isomers, and 2-Cl-diphenidine (2-Cl-DPH) were investigated using binding studies at 46 central nervous system receptors including the *N*-methyl-D-aspartate receptor (NMDAR), serotonin, dopamine, norepinephrine, histamine, and sigma receptors as well as the reuptake transporters for serotonin, dopamine and norepinephrine. Reuptake inhibition potencies were measured at serotonin, norepinephrine and dopamine transporters. NMDAR antagonism was established *in vitro* using NMDAR-induced field excitatory postsynaptic potential (fEPSP) experiments. Finally, DPH and 2-MXP were investigated using tests of pre-pulse inhibition of startle (PPI) in rats to determine whether they reduce sensorimotor gating, an effect observed with known dissociative drugs such as phencyclidine (PCP) and ketamine. The results suggest that these 1,2-diarylethylamines are relatively selective NMDAR antagonists with weak off-target inhibitory effects on dopamine and norepinephrine reuptake. DPH and 2-MXP significantly inhibited PPI. DPH showed greater potency than 2-MXP, acting with a median effective dose (ED_50_) of 9.5 mg/kg, which is less potent than values reported for other commonly abused dissociative drugs such as PCP and ketamine.

## Introduction

1,2-Diarylethylamines represent a structural class of organic molecules, which all share a core structure comprised of an ethylamine nucleus with vicinal aromatic substitutions. These compounds have diverse pharmacology and modifications of this structure have yielded analgesics, antidepressants, anticonvulsants and neuroprotective agents [1–3]. Their pharmacology appears to be mediated through a range of interactions including activation of opioid receptors [2, 4, 5], inhibition of monoamine transporters [6, 7] and antagonism of glutamatergic *N*-methyl-D-aspartate receptors (NMDARs) [1, 8]. Clinical interest in 1,2-diarylethylamines includes the well-tolerated NMDAR antagonist lanicemine, which exhibits antidepressant activity[1], and remacemide, which has shown promise in clinical trials for several therapeutic areas including neurodegenerative diseases, epilepsy and stroke [3]. Structures of some of these are given as supporting information (S1 Fig).

Most recently, a variety of 1,2-diarylethylamines have appeared as ‘legal highs’ or ‘research chemicals’ from online vendors. These include the opioid MT-45 [9, 10] and several dissociative agents such as diphenidine (DPH) and 2-methoxydiphenidine (methoxphenidine or 2-MXP) [11–13]. The dissociative diarylethylamines emerged as ‘legal highs’ or ‘research chemicals’, to circumvent regulations on human consumption, shortly following a United Kingdom ban (February 2013) on arylcyclohexylamine-based dissociative drugs such as methoxetamine (MXE) and 3-methoxyphencyclidine (3-MeO-PCP) [13, 14]. Though DPH and 2-MXP were new to this market and had no previously documented history of human use, syntheses had been published as early as 1924 and 1989 respectively and both had undergone *in vitro* screening for NMDAR affinity.[13] Images of products sold online are provided as supporting information (S11–S13 Figs).

The phenomenology of the altered state induced by dissociative drugs is complex and dose dependent. However, key features of the dissociative state include sensory hallucinations, tactile distortions, euphoria, derealization and depersonalization [13]. A significant portion of the therapeutic and psychoactive effects of dissociative drugs is believed to be mediated through NMDAR antagonism [13, 15, 16]. Although NMDAR antagonism appears to be a common denominator involved in the dissociative pharmacology, additional receptors are likely to contribute to the effects of individual compounds [16].

Aside from some NMDAR binding studies [17, 18] and a recent publication about the metabolism of DPH [19], little information has been published regarding the pharmacology of the dissociative ‘research chemicals’ DPH and 2-MXP. Due to the increasing appearance of 1,2-diarylethylamine based ‘research chemicals’ [11–13] along with reports of overdoses [20] and fatal intoxications [21] it is important to investigate the pharmacology of these compounds.

**Fig 1:**
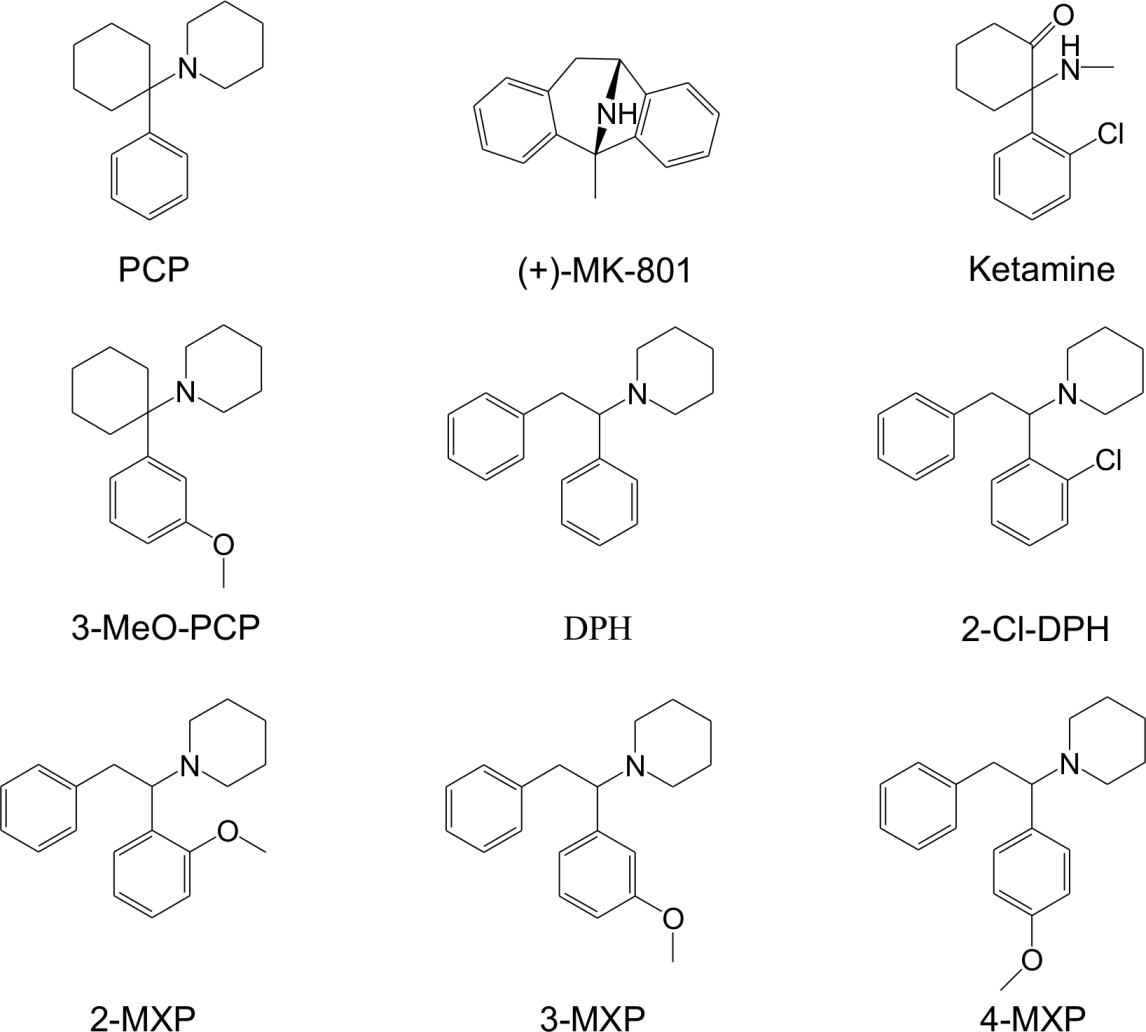
Structures of PCP and related arylcyclohexylamines, ‘legal highs’ DPH, 2-MXP and aryl-substituted 1,2-diarylethylamines.

To extend earlier reports of NMDAR binding, competitive binding experiments with [^3^H]-MK-801 were performed with DPH and 2-MXP, along with the methoxy- substituted positional isomers 3-methoxy-diphenidine (3-MXP) and 4-methoxy-diphenidine (4-MXP) as well as 2-Cl-diphenidine (2-Cl-DPH) (Fig 1). The NMDAR antagonists, PCP, ketamine, (+)-MK-801 and memantine served as reference compounds. NMDAR selectivity was investigated using binding studies at an additional 45 CNS receptor sites including G protein-coupled receptors (serotonin, dopamine, norepinephrine, histamine, acetylcholine subtypes), monoamine reuptake transporters for dopamine (DAT), norepinephrine (NET) and serotonin (SERT), mu (MOR), kappa (KOR) and delta (DOR) opioid receptors and sigma-1 and sigma-2 receptor sites. Compounds were also evaluated for inhibition of monoamine reuptake to establish the functional consequences of the observed interactions with monoamine reuptake transporters. To measure functional activity at central synapses *in vitro*, the target compounds along with reference compounds were assessed on NMDA receptor-mediated field excitatory postsynaptic potentials (NMDAR-fEPSP). Finally, *in vivo* pre-pulse inhibition (PPI) experiments were performed with DPH and 2-MXP.

## Materials and Methods

### Target Compounds

Phencyclidine (PCP), memantine hydrochloride and (+)-MK-801 maleate were obtained from Sigma-Aldrich.

Synthesis and analytical characterizations of the target 1,2-diarylethylamines have been published elsewhere [11, 12]. The exception is 2-Cl-DPH, which was not described previously. Details of the synthesis and analytical characterization of 2-Cl-DPH are provided as supporting information (S6 File).

### NMDA Receptor Binding Studies

*In vitro* binding affinities (K_i_) of the target compounds at the PCP site within the NMDAR channel were determined using competitive radioligand binding studies with [^3^H]-MK-801 in accordance with established protocols published by Reynolds and Sharma [22, 23]. Thoroughly washed rat forebrain homogenate were used as the NMDAR source (whole brain obtained from Pel-Freez Biologicals) and prepared as described by Reynolds and Sharma [22]. Suspensions of 10 mM HEPES buffer (pH 7.4 at room temperature) containing 100 μg/mL protein, 1 nM (+)-[^3^H]-MK-801, 100 μM glutamate, 10 μM glycine, and various concentrations of unlabeled competitor or 30 μM (+)-MK-801 for nonspecific binding (and positive control), were incubated in the dark on a mechanical rocker at room temperature for 2 h. The reaction was terminated by vacuum filtration using a 24 well cell harvester (Brandel, Gaithersburg, MD) over presoaked GF/B glass fiber filters (Brandel, Gaithersburg, MD). Filters were washed with room temperature assay buffer (3 x 5 mL). Tritium trapped on the filter was measured via liquid scintillation counting, using a Beckman LS 6500 multipurpose scintillation counter (BeckmanCoulter, USA) at 57% efficiency. IC_50_ values were determined in Graphpad Prism 5.0 using non-linear regression with log-concentration plotted against percent specific binding. Percent specific binding for [^3^H]-MK-801 in a control experiment was ~95%. K_i_ values were calculated using the equation of Cheng and Prusoff [24]. The K_d_ for (+)-MK-801 (1.747 nM), was determined via homologous binding assay as described by Reynolds and Sharma and is consistent with the literature [22]. Protein concentration was determined via the Bradford method using Coomassie protein assay reagent (Sigma, USA) [25] with rat albumin (Sigma, USA) as standard. Experiments were performed in duplicate and repeated a minimum of three times. Raw counts per minute (CPM) for all NMDAR binding experiments are provided as supporting information (S1 File).

### Non-NMDA Receptor Binding Studies

Radioligands and concentrations used for additional 45 CNS receptor binding assays are listed as supporting information (S1 Table). This work was performed by the National Institute of Mental Health Psychoactive Drug Screening Program (NIMH PDSP) as described previously.[26] Briefly target compounds were dissolved in DMSO and subjected to a primary screen at 10,000 nM concentration. Compounds exhibiting >50% inhibition were subjected to a secondary assay at varying concentrations to determine K_i_ values. Additional experimental details are available in the NIMH PDSP assay protocol book [27].

### Monoamine Reuptake Inhibition Assays

Monoamine reuptake inhibition assays were performed via the NIMH PDSP as previously described.[28] In brief, the neurotransmitter transporter uptake assay kit (R8174) (Molecular Devices) was used. Human monoamine transporters (DAT, NET and SERT) were stably expressed in HEK293 cells. Assay buffer consisted of 20 mM HEPES, 1x HBSS at pH 7.4. Cells were plated in Pol-L-Lys (PLL) coated 384-well black clear bottom cell culture plates with a density of 15,000 cells/well. Cells were incubated for at least 6 h prior to assay. Media was removed and replaced with 20 μL assay buffer and 5 μL of drug or positive control (cocaine or nisoxetine) as a 5X stock. Cells were incubated for 30 min at 37°C. 25 μL of dye solution was then added and following an additional 30 min incubation (at 37°C) fluorescence intensity was measured using a FlexStation II (bottom read mode, excitation wavelength = 440 nM, emission = 520 nM with a cutoff of 510 nM). Relative florescence units were exported and plotted against drug concentration to obtain 50% inhibition potencies (IC_50_) using non-linear regression (Prism 5.0). Additional details are available in the NIMH PDSP assay protocol book [27].

### *In Vitro* Field Excitatory Postsynaptic Potential (fEPSP) Experiments

Male Wistar rats (Crl:Wi; Charles River, UK) aged 9–10 wk were sacrificed by neck vertebral dislocation (schedule 1 method) according to the United Kingdom (Scientific Procedures) Act of 1986. After their rapid removal, brains were placed in artificial cerebrospinal fluid (aCSF) consisting of 124 mM NaCl, 26 mM NaHCO_3_, 3 mM KCl, 1.4 mM NaH_2_PO4, 1 mM MgSO_4_, 2 mM CaCl_2_, and 10 mM D-glucose and continuously oxygenated with 95% O_2_ and 5% CO_2_. The brain was cut parasagittally into 400 μm sections and hippocampal slices were removed, stored and placed in a submerged recording chamber at 28–30°C. Recordings of synaptic activity were made, analyzed and presented as described [29, 30]. A bipolar electrode was used to deliver stimuli (0.03 Hz) to the Schaffer collateral pathway to enable recording of field excitatory synaptic potentials (fEPSPs) using a glass microelectrode positioned in the stratum radiatum of area CA1. The NMDA receptor-mediated component of the fEPSP (NMDAR-fEPSP) was revealed by adding 10 μM NBQX, 50 μM picrotoxin and 1 μM CGP 55845 to the aCSF, which abolished AMPA and GABA receptor mediated transmission. After 30 min of stable control responses, 1 and 10 μM solutions of the individual compounds were added to the perfusate for 3–12 h while recording the amplitude and area of the NMDAR-fEPSP on-line using WinLTP [31]. Single exponential curves were fitted to the graphs and curves extrapolated to show likely half-times and minimum plateau responses achieved with each concentration of the compounds. Raw data for all fEPSP experiments are provided as supporting information (S2, S3 and S4 Files).

## *In Vivo* Pre-pulse Inhibition (PPI) Experiments

### Animals

Male Sprague–Dawley rats from Harlan Industries (Indianapolis, IN, USA; initial weight 250–275 g) were housed in pairs under a 12-h reverse light/dark cycle (lights off at 0700 h). Use of a reversed light/dark cycle allows behavioral testing to be conducted during the awake phase of the light/dark cycle. Food and water were available *ad libitum*. Animals were acclimatized for approximately 1 wk after arrival prior to behavioral testing and maintained in Association for Assessment and Accreditation of Laboratory Animal Care (AAALAC)-approved facilities that meet all federal and state guidelines. Procedures were approved by the University of California San Diego institutional animal care and use committee. Principles of laboratory animal care were followed.

### Drugs

DPH hydrochloride and 2-MXP hydrochloride were dissolved in water containing 5% Tween-80 and administered subcutaneously, 10-min prior to the start of the startle test session. The injection volume was 1 ml/kg.

### Acoustic startle apparatus and test sessions

#### Apparatus

Eight startle chambers (SR-LAB system, San Diego Instruments, San Diego, CA, USA) were used to measure startle reactivity in rats [32, 33]. The startle test chambers were sound-attenuated, lighted, and ventilated enclosures containing a clear nonrestrictive cylindrical Plexiglas stabilimeter, 8.2 cm in diameter. A high-frequency loudspeaker mounted 24 cm above the Plexiglas cylinder produced all acoustic stimuli. The peak and average amplitudes of the startle response were detected by a piezoelectric accelerometer. At the onset of the startling stimulus, 100 1-ms readings were recorded, and the average amplitude was used to determine the magnitude of the startle response (measured in arbitrary units). A dynamic calibration system was employed to ensure comparable stabilimeter sensitivity across test chambers, and sound levels were measured using the dB(A) scale.

#### Test sessions

Acoustic startle test sessions consisted of startle trials (pulse-alone) and prepulse trials (prepulse + pulse). The pulse-alone trial consisted of a 40-ms 120-dB pulse of broadband white noise. Prepulse + pulse trials consisted of a 20-ms acoustic prepulse, an 80-ms delay, and then a 40-ms 120-dB startle pulse (100-ms onset–onset). There was an average of 15 s (range = 6–22 s) between trials. During each inter-trial interval, the movements of the animals were recorded once to measure response when no stimulus was present (data not shown). Each startle session began with a 5 min acclimation period to a 65-dB broadband noise that was present continuously throughout the session. One week after arrival, animals were tested in a brief baseline startle/PPI session to create treatment groups matched for levels of startle and PPI. The startle test session contained 12 pulse-alone trials and 36 prepulse + pulse trials (12 prepulses each of 68-, 71-, and 77-dB [equivalent to 3-, 6-, and 12-dB above background]) presented in a pseudo-randomized order. Six pulse-alone trials were presented at the beginning and the end of the test session but were not used in the calculation of PPI values. Raw data for all PPI experiments are provided as supporting information (S5 File).

#### Analysis

The amount of PPI was calculated as a percentage score for each prepulse + pulse trial type: %PPI = 100−{[(startle response for prepulse + pulse trial)/(startle response for pulse-alone trial)] × 100}. Startle magnitude was calculated as the average response to all of the pulse-alone trials. PPI data were analyzed with two-factor analysis of variance (ANOVA) with treatment as the between-subjects factor and trial type (prepulse intensity) as a repeated measure. For experiments in which there was no significant interaction between drug and prepulse intensity, PPI data were collapsed across prepulse intensity and average PPI was used as the main dependent measure. ED_50_ values were calculated using nonlinear regression. Startle magnitude data were analyzed with one-factor ANOVA. Post-hoc analyses were performed using Tukey’s studentized range method. The alpha level was set at 0.05.

## Results and Discussion

### Receptor Binding affinities

NMDAR binding affinities for the test compounds are presented in Table 1. [^3^H]-MK-801 displacement curves for the five 1,2-diphenylethylamines and reference compounds are provided as supporting information (S3 and S4 Figs respectively). Four of the five compounds had potent nM affinity for [^3^H]-MK-801 labeled NMDARs. 4-MXP had lower NMDAR affinity (461 nM) compared to the other members of the series. The rank order of potency of DPH and its three MeO- isomers (DPH = 3-MXP > 2-MXP > 4-MXP) is comparable with that seen with the equivalently substituted arylcyclohexylamines; with PCP = 3-MeO-PCP > 2-MeO-PCP > 4-MeO-PCP [34]. The parallel structure activity relationships (SAR) between aryl-methoxy substituted derivatives of PCP and DPH is not surprising given the structural overlap between the benzyl-piperidine portions of PCP and DPH (Fig 1). Therefore, the overlapping portions of arylcyclohexylamines and 1,2-diphenethylamines likely bind to the same region of the PCP binding site within the NMDAR channel. SAR studies on arylcyclohexylamines may have relevance for further optimization of 1,2-diarylethylamine-based NMDAR antagonists.

**Table 1:** NMDAR binding affinities for five target 1,2-diphenylethylamines and reference compounds

Compound	IC_50_ ± SEM (nM)	K_i_ ± SEM (nM)
**PCP [34]**	91 ± 1.3	57.9 ± 0.8
**Ketamine**	508.5 ± 30.1	323.9 ± 19.2
**Memantine**	594.2 ± 41.3	378.4 ± 26.3
**(+)-MK-801**	4.1 ± 1.6	2.5 ± 1.0
**DPH**	28.6 ± 3.5	18.2 ± 2.2
**2-MXP**	56.5 ± 5.8	36.0 ± 3.7
**3-MXP**	30.3 ± 2.6	19.3 ± 1.7
**4-MXP**	723.8 ± 69.9	461.0 ± 44.5
**2-Cl-DPH**	14.6 ± 2.1	9.3 ± 1.3

NMDAR binding affinities determined using [^3^H]-(+)-MK-801 in rat forebrain. Mean ± SEM are presented from four separate experiments run in duplicate.

Binding affinities for the test compounds at NMDAR were described previously in a patent by Gray and Cheng [17]. Notably, in most cases the NMDAR binding affinities reported herein differed from those earlier studies. The rank order of potencies were, however, consistent. Surprisingly, Gray and Cheng reported 2-Cl-DPH to have pM affinity, an order of magnitude higher than other known NMDAR antagonists. This report of high affinity binding reported for 2-Cl-DPH prompted us to reinvestigate this compound in the present study. While in the current study 2-Cl-DPH had potent low nM affinity for NMDAR (9.3 nM), this is substantially less than the pM affinity reported previously. In a study by Berger *et al*., the two DPH enantiomers showed lower affinity than that reported both here and by Gray and Cheng ((S-)-DPH 120 nM, (R)-DPH 5,250 nM) [18]. Likewise, the reported affinity of PCP was ~4-fold lower than that observed here and reported by others [35]. These discrepancies may result from the fact that [^3^H]-TCP was used by Gray and Cheng while the more selective [^3^H]-MK-801 [36–39] was used in the current study. Berger *et al*. used [^3^H]-MK-801 to label NMDAR from a different tissue source (whole cell membranes prepared from rat cortex and hippocampus) than the current study (rat forebrain homogenate) [18]. Differences in affinities of the compounds for different NMDAR subunit combinations might be one possible explanation.

### Non-NMDA Receptor Binding Affinities

**Fig 2:**
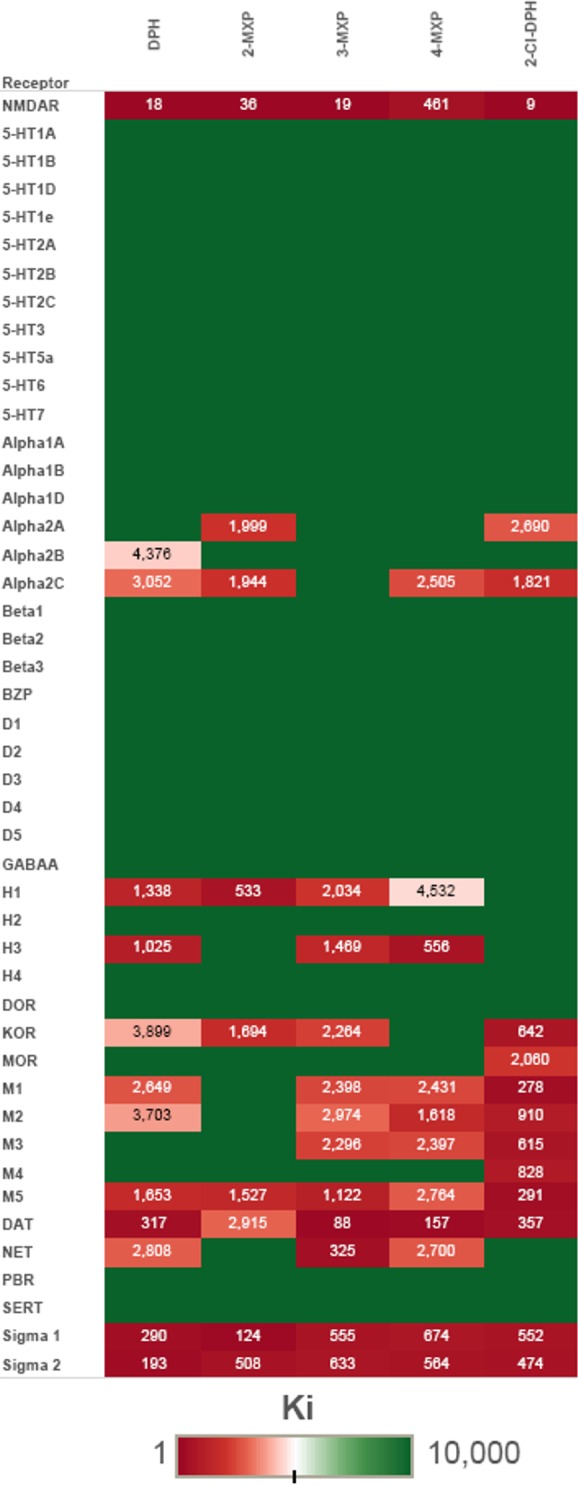
Heat map of receptor binding affinities observed for NMDAR and 45 additional receptor sites. K_i_ values given in nM. Full green without numbers represents >10,000 nM in the primary screen.

All the target compounds displayed lower affinities at all other CNS sites evaluated relative to NMDAR with the exception of 4-MXP, the weakest NMDAR ligand, which had a ~2.5-fold higher affinity at DAT than NMDAR. A heat map of binding results is presented in Fig 2, which also shows experimentally determined K_i_ values for those receptors as well as for NMDAR.

Most of the target compounds had weak affinity for the KOR whereas only 2-Cl-DPH showed weak affinity (2,060 nM) for MOR. These results are consistent with the fact that several related 1,2-diarylethylamines including lefetamine, AD-1211 and MT-45 (S1 Fig) act as opioid agonists and analgesics [2, 4, 5, 40]. However the lack of high affinity suggests that the 1,2-diarylethylamines evaluated here do not act as opioids. Interactions with muscarinic receptor subtypes (M1, M2, and M5) were generally weak. 2-Cl-DPH had the highest affinities of the series for muscarinic sites, although affinities at these sites were 30-fold less than for NMDAR. Most of the target compounds had submicromolar affinity for DAT, with 3-MXP exhibiting the highest affinity (81 nM) followed by 4-MXP (157 nM), DPH (317 nM), 2-Cl-DPH (357 nM) and finally 2-MXP which had the weakest affinity (2,915 nM). Several target compounds also had low affinity for NET, while notably none of the target compounds showed affinity for SERT. The absence of SERT affinity was surprising in light of reports indicating that 1,2-diphenylethylpiperazines act as norepinephrine/serotonin reuptake inhibitors with low DAT affinity [6, 7]. Notably, in addition to having a piperazine ring in place of piperidine, most of the 1,2-diphenylpiperzines investigated [6, 7] were substituted on the 2-phenyl ring as opposed to the current compounds, which were substituted on the 1-phenyl ring. How substitutions on the 2-phenyl ring influence NMDAR affinity or the psychoactive effects of 1,2-diarylethylamines warrants investigation. A number of arylcyclohexylamines, including MeO-PCP isomers, have affinity at monoamine reuptake transporters [34, 35]

Finally, moderate affinities were seen for the sigma-1 and sigma-2 receptors. Many arylcyclohexylamines, as well as other NMDAR antagonists, have high affinity for sigma receptors [34]. The significance of these sigma receptor interactions on the effects of NMDAR antagonists is still unknown, although sigma receptors have been implicated in the regulation of a large number of diverse neurotransmitter and physiological systems including dopaminergic and glutamatergic neurotransmission (particularly sigma-1) [41–43], intracellular calcium (sigma-1 and sigma-2) [43, 44] and may play roles in abuse liability and tolerance [43].

#### Monoamine reuptake inhibition

The five diarylethylamines were evaluated in monoamine reuptake inhibition assays. Functional potencies (IC_50_) of the compounds for reuptake inhibition at DAT, NET and SERT are given in Table 2. Consistent with the lack of SERT affinity in the binding assays, most of the target compounds failed to show functional activity when screened at 10 μM. Only 4-MXP displayed weak reuptake inhibition (IC_50_ = 19.0 μM) at SERT. Overall there was good agreement between the functional potency (IC_50_) in the uptake assays and receptor binding affinities (K_i_) for DAT and NET. For example, the most potent reuptake inhibitor evaluated, 3-MXP, also had the highest affinities at DAT and NET. It is unknown whether monoamine reuptake inhibition contributes to the pharmacology of diarylethylamines *in vivo*. Interestingly, many arylcycloalkylamines also have been reported to have affinity at DAT, NET and SERT [34, 35]. Dual inhibition of NMDAR and monoamine reuptake may provide clinically relevant polypharmacology with therapeutic potential in a number of areas, including neurodegenerative diseases, epilepsy and depression.

**Table 2:** Inhibition potencies of 1,2-diarylethylamines as monoamine transporter reuptake inhibitors.

IC_50_ (μM)			
Compound	DAT	NET	SERT
**DPH**	1.99 (0.91)	9.25 (0.97)	>10 μM
**2-MXP**	30.0 (0.81)	35.2 (2.04)	>10 μM
**3-MXP**	0.587 (0.92)	2.71 (0.95)	>10 μM
**4-MXP**	2.23 (0.96)	22.5 (1.75)	19.0 (1.12)
**2-Cl-DPH**	10.5 (0.65)	27.1 (1.02)	>10 μM

IC_50_ values shown in μM. Hill slopes shown in parenthesis. ND–IC_50_ values were not determined because compounds showed less than 50% inhibition of uptake at 10 μM during a preliminary screening.

#### Effect on NMDA receptor-mediated field EPSPs

**Fig 3:**
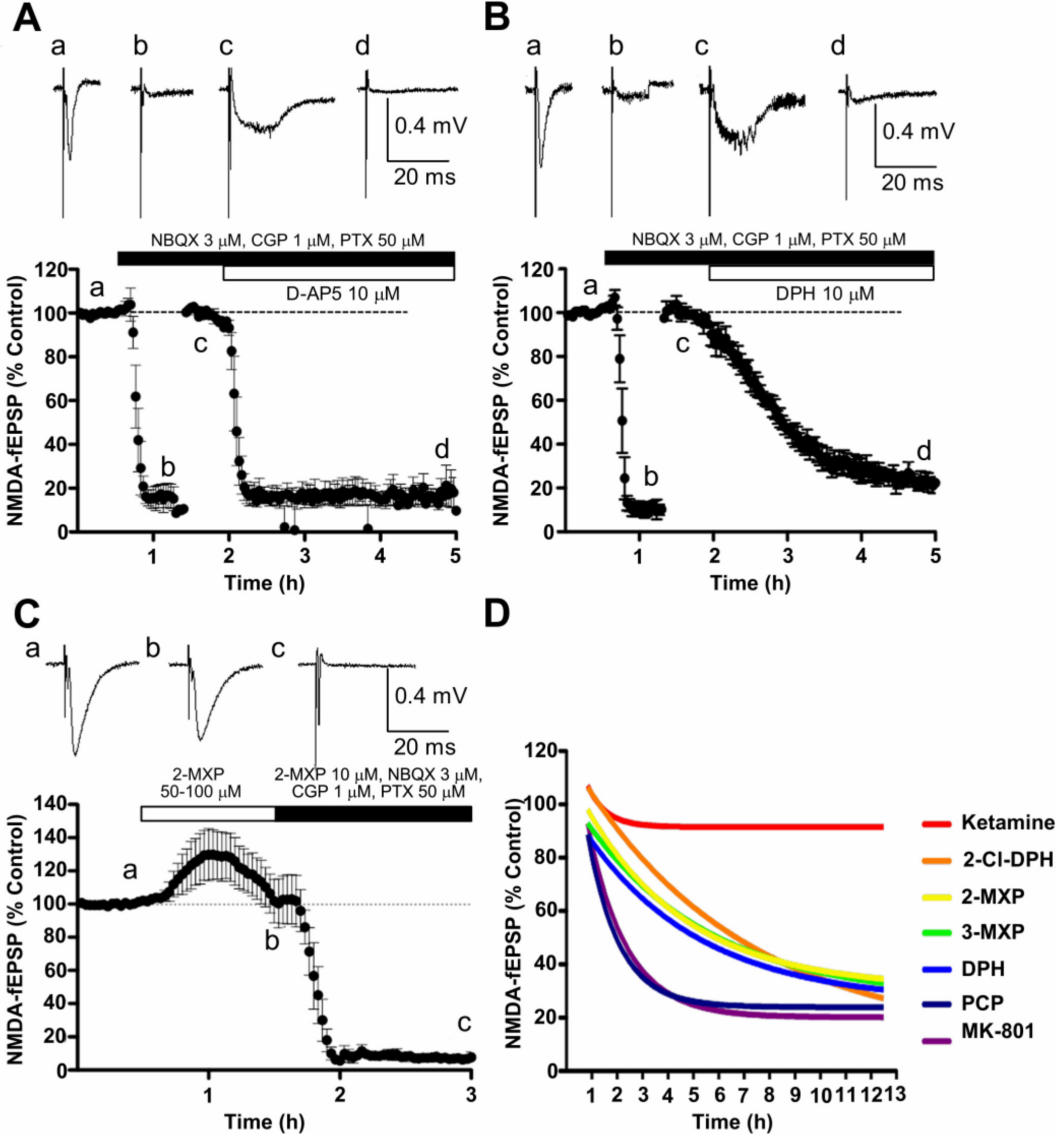
Comparison of effect of compounds on hippocampal fEPSPs. A & B. Graphs showing basic method for studying effect of compounds on NMDAR-fEPSPs in CA1 region comparing effects of 10 μM D-AP5 (A) and DPH (B). After 30 min baseline recording (a), AMPA and GABA receptor mediated events were blocked pharmacologically (b), the stimulus strength increased and a new baseline was obtained for the NMDAR-fEPSP (c) before administering the test compound for 3 h (d). Note firstly the difference in shape between the AMPA receptor-mediated (a) and the NMDA receptor-mediated (c) fEPSPs, and secondly the slow onset of the pharmacological block by DPH (B) compared with D-AP5 (A). C. AMPA receptor-mediated fEPSPs (a) were not reduced by 50/100 μM 2-MXP (b) but the remaining NMDAR-fEPSPs after AMPA and GABA receptor antagonism were abolished in the presence of 10 μM 2-MXP(c). D. Superimposed single exponentials, showing time course of reduction of NMDAR-fEPSP by 1 μM test compounds superfused for up to 12 h.

Fig 3 shows the effects of target and reference compounds on fEPSPs recorded from CA1 neurons in hippocampal slices following Schaffer collateral stimulation. In Fig 3A and 3B, a cocktail of compounds was used to block the AMPA and GABA receptor-mediated events and the stimulus strength was increased to provide a submaximal NMDAR-fEPSP. Fig 3A shows the rapid and near maximal effect of the competitive NMDAR antagonist, D-AP5 (10 μM). The small persistent deflection, measured as <20%, is likely to represent a combination of non-glutamatergic synaptic transmission and stimulus artifact.

Using the same experimental design, DPH 10 μM produced a similar but much slower reduction of the NMDAR-fEPSP. Equilibrium was not achieved during the 3 h superfusion (Fig 3B).

**Fig 4:**
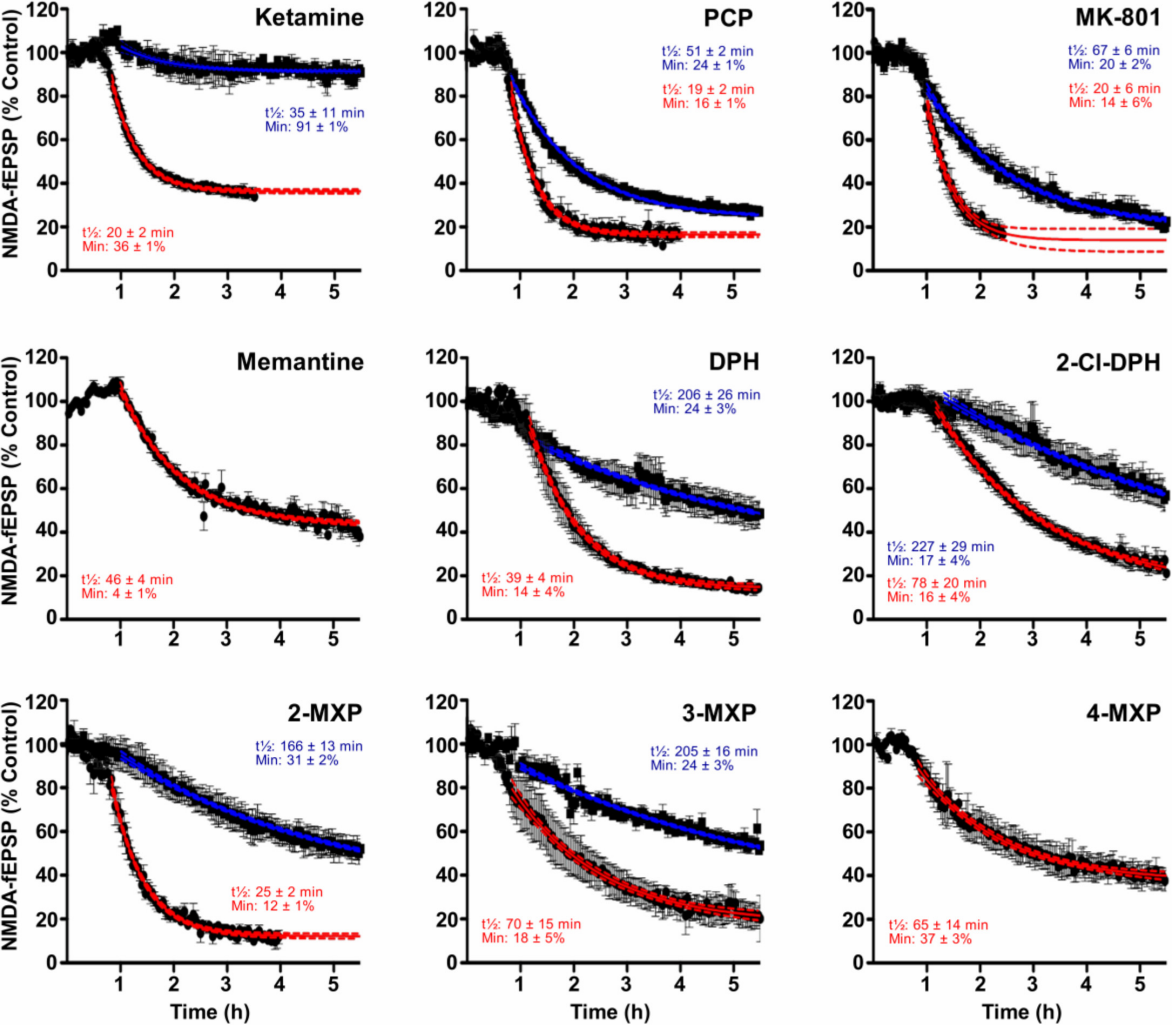
Graphs showing the time course of reduction of the NMDAR-fEPSPs by nine compounds. Each graph shows the average fEPSP amplitude of 3–5 experiments expressed as a percentage of the amplitude during the prior baseline recording. Drugs were added to the aCSF at concentrations of either 1 μM (blue) or 10 μM (red). The figures on the graphs give i) the time to reach half the maximal response for each drug concentration and ii) the maximal effect; values derived from single exponential curve-fitting. In the case of memantine and 4-MXP only the 10 μM concentration was tested.

Using the same protocol as in Fig 3B, nine compounds were initially tested at 10 μM; all reduced the NMDAR-fEPSP recorded from CA1 neurones following Schaffer collateral stimulation. In all cases, the NMDAR-fEPSP declined slowly in the presence of both the DPH analogues and the established channel blocking NMDAR antagonists (Fig 4). Only for ketamine, memantine and 4-MXP was a submaximal plateau level of inhibition achieved. Similarly at 1 μM for most of the compounds, the slow kinetics did not allow submaximal plateau responses to be reached in a practical time period which makes estimating potency difficult (Fig 4). Even 12 h was not long enough to achieve maximal effects, as was found for example with 1 μM DPH, so exponential plots (see Fig 3D), were used to estimate the half-time and the minimum plateau values achieved by concentrations of 1 and 10 μM, and 0.3 μM for MK-801 (data not shown). Relative potencies of the compounds were approximated from rates of antagonism and the plateau levels reached. Ranking of these relative potencies (MK-801 > PCP > 2-Cl-DPH ≥ DPH ≥ 3-MXP ≥ 2-MXP > Ketamine > 4-MXP ≥ memantine) correlated with relative potencies in binding assays, which gave a Spearman’s rank correlation coefficient of 0.78 (p = 0.0172, two-tailed).

To examine the specificity of DPH analogues, 2-MXP was superfused at 50 (n = 2) and 100 μM (n = 1) in the absence of AMPA and GABA receptor antagonists (Fig 3C). No reduction of the AMPA receptor-mediated fEPSP was seen, rather, there was a small increase, which may represent reduced NMDAR-mediated excitation of inhibitory interneurons. These data suggest that 2-MXP was >50 times more potent as an antagonist of NMDAR than of AMPA receptor responses.

Unlike D-AP5 and ketamine, no signs of recovery from antagonism with the 1,2-diarylethylamines were seen during 2 h of superfusion in control aCSF (preliminary observations–not illustrated). Such slow *in vitro* kinetics are more reminiscent of MK-801 than of ketamine, as illustrated here (Fig 3D).

### *In Vivo* Pre-pulse Inhibition Experiments

The startle response is attenuated if the startle-inducing stimulus is preceded by a weak subthreshold prepulse. This phenomenon, known as prepulse inhibition (PPI), serves as an operational measure of sensorimotor gating, a type of pre-attentional sensory filtering [45]. NMDAR antagonists with dissociative effects, including PCP, ketamine, methoxetamine, MK-801, and dextrorphan, produce disruptions of PPI in rats [32, 33, 46]. The dissociative and hallucinogenic effects of NMDAR antagonists are believed to reflect a loss of subcortical filtering mechanisms, resulting in cortical sensory overload [47]. Hence, the PPI disruption produced by NMDAR antagonists may reflect the information processing deficits that contribute to their dissociative effects.

**Fig 5:**
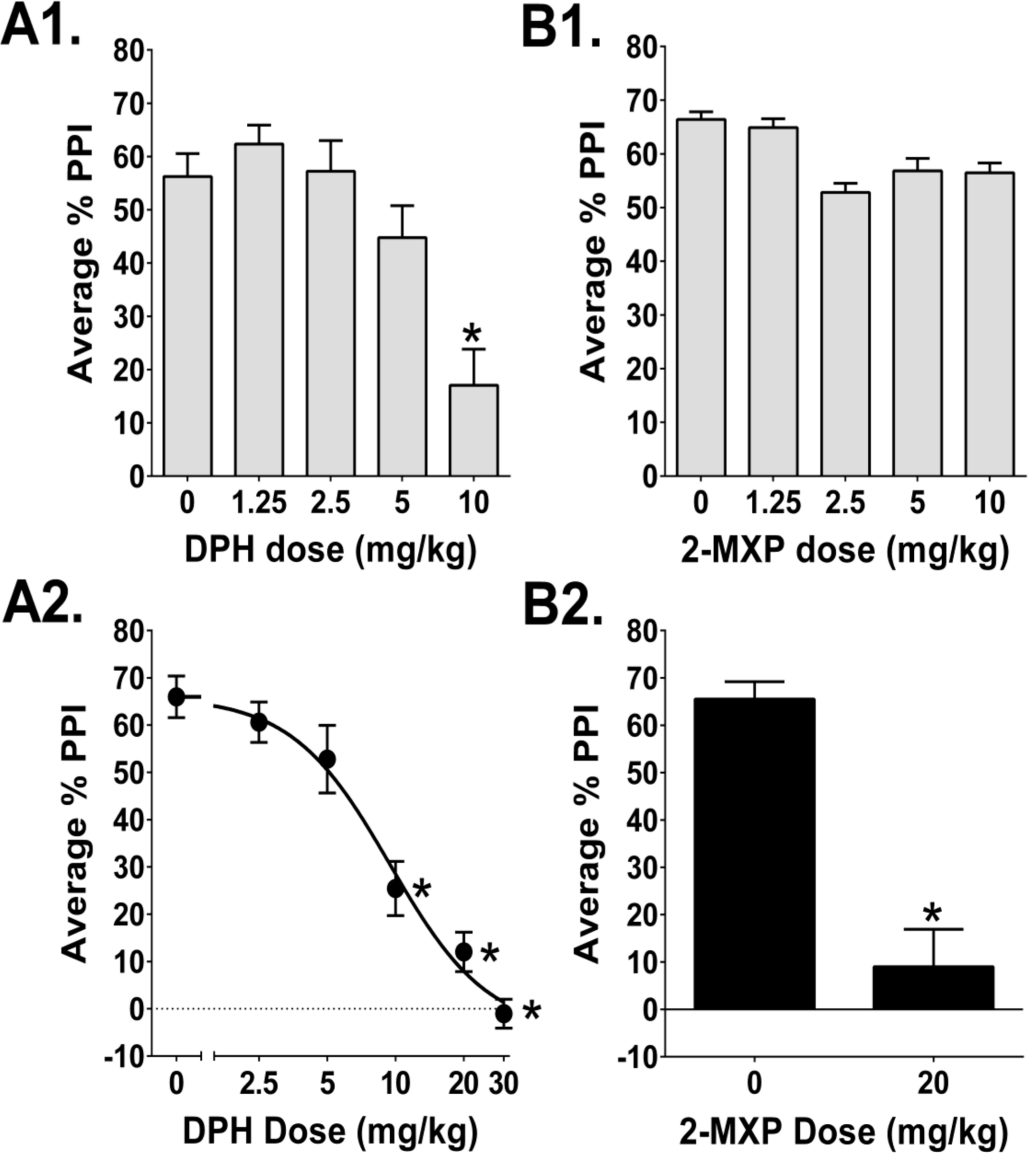
**Effects of (*A*) DPH and (*B*) 2-MXP on prepulse inhibition (PPI**). (*A1*) Effect of DPH at doses ranging from 1.25–10 mg/kg. 50 rats were used (*n* = 10/group). (*A2*) Effect of DPH at doses ranging from 2.5–30 mg/kg. 49 rats were used (*n* = 9-10/group). (*B1*) Effect of 2-MXP at doses ranging from 1.25–10 mg/kg. 50 Rats were used (*n* = 10/group). (*B2*) Effect of 20 mg/kg 2-MXP. 24 rats were used (*n* = 12/group). PPI values shown are averaged across the 3 prepulse intensities (mean ± SEM). *Significant difference compared with vehicle control, *p*<0.01 (Tukey's test).

DPH significantly reduced PPI (*F*(4,45) = 11.33, *p*<0.0001; Fig 5A1). PPI was assessed using 3 prepulse intensities (68, 71, and 78-dB above background), but there was no interaction between treatment and prepulse intensity (*F*(8,90) = 1.89, *NS*), so the PPI data were collapsed across the three prepulse intensities. Administration of 10 mg/kg DPH significantly reduced average PPI (*p*<0.01, Tukey's test). There was a significant main effect of prepulse intensity in this experiment (*F*(2,90) = 93.17, *p*<0.0001) and in all subsequent tests (data not shown).

Similar to the first dose-response experiment, DPH significantly reduced PPI at higher doses (*F*(5,54) = 31.06, *p*<0.0001; Fig 5A2). Although here there was an interaction between treatment and prepulse intensity (*F*(10,108) = 7.30, *p*<0.0001), the 10, 20, and 30 mg/kg doses of DPH reduced PPI at all three prepulse intensities (*p*<0.01, Tukey’s test). DPH reduced average PPI with an ED_50_ = 9.5 mg/kg (95% CI 5.9–15.1 mg/kg), which is equivalent to 31.5 (19.5–50.0) μmol/kg.

In contrast to the results with DPH, 2-MXP did not alter PPI at doses up to 10 mg/kg (Main effect: *F*(4,44) = 1.03, *NS*; Fig 5B1). When tested at 20 mg/kg, however, 2-MXP did disrupt PPI (Main effect: *F*(1,22) = 41.93, *p*<0.0001). Unfortunately, it was not possible to fully characterize the dose-response of 2-MXP because of the limited amount of drug available for testing.

Administration of 20 mg/kg 2-MXP significantly reduced the acoustic startle response (ASR) from 175.9 ± 29.2 to 78.8 ± 11.9 (Main effect: *F*(1,22) = 9.55, *p*<0.0053). To confirm whether the effect of 2-MXP on PPI was independent of changes in the magnitude of the ASR, we examined the effect on PPI in subgroups of animals matched for startle level.[48] This was an important control because large reductions in ASR can potentially impair PPI due to floor effects; therefore, it was important to determine whether drug effects on PPI and ASR were independent. Eight animals with overlapping startle responses were chosen from each group (mean ± SEM = 128.5 ± 14.4 and 100.7 ± 10.1). Though 2-MXP had no effect on ASR magnitude in those subgroups (*F*(1,14) = 2.49, *NS*), PPI was still significantly reduced (Main effect of 2-MXP: *F*(1,14) = 53.47, *p*<0.0001). Hence, the PPI disruption produced by 2-MXP was not caused by changes in ASR magnitude. In contrast to 2-MXP, DPH did not alter the magnitude of the ASR in either experiment (*F*(4,45) = 0.95, *NS*; *F*(5,54) = 0.82, *NS*).

An unexpected observation was the fact that DPH and 2-MXP were less potent in the PPI experiments than might be anticipated based on their high NMDAR affinities. Compared with DPH, PCP displaced [^3^H]MK-801 with 3-fold lower affinity, but altered PPI with 10-fold higher potency (PCP disrupts PPI with an ED_50_ = 3.15 μmol/kg [49]). Although DPH binds to the NMDAR with ~18-fold higher affinity than ketamine (Table 1), DPH was substantially less potent than ketamine enantiomers than DPH in PPI experiments in rats.[49]

Likewise, anecdotal reports in humans [13] suggest DPH and 2-MXP may be less potent than ketamine. Follow-up research is required to address this discrepancy. The pharmacokinetics of 1,2-diphenylethylamines may play a role in their relatively low potency in rats. It is possible that 1,2-diphenylethylamines are substrates for P-glycoprotein transporters or are more rapidly metabolized than arylcyclohexylamines. In rats, DPH is metabolized by hydroxylation, dehydrogenation, and *N*-dealkylation [19]. The possibility that pharmacokinetic factors may reduce the potency of DPH and 2-MXP has important clinical implications, particularly in regards to drug-drug interactions.

## Conclusion

Five 1,2-diarylethylamines including the ‘legal highs’ DPH and 2-MXP, were found to be relatively selective NMDAR antagonists. Some off-target interactions were observed and several compounds also showed moderate nM to μM activity as inhibitors of dopamine and norepinephrine reuptake with selectivity over serotonin transport. These compounds also inhibited NMDAR-mediated field EPSPs with a rank-order similar to their measured NMDAR affinities. Notably, the compounds showed a slow onset of inhibition which may have significance for their subjective effects in humans. Finally, DPH was found to reduce average PPI with an ED_50_ = 9.5 mg/kg (95% CI 5.9–15.1). 2-MXP showed a significant inhibition of PPI at 20 mg/kg but not 10 mg/kg. Thus, DPH and 2-MXP are less potent in *in vivo* PPI experiments than other NMDAR antagonists like PCP and ketamine, a finding that was not anticipated based on their measured NMDAR affinities. Taken together, the presented results are consistent with the dissociative effects of these compounds anecdotally reported by human users. These results hold significance for potential therapeutic use, governmental regulation and harm reduction strategies.

## Supporting Information

S1 Fig Structures of some clinically investigated 1,2-diarylethylamines. (TIF)

S2 Fig Receptor binding for reference compounds showing displacement of [^3^H]-MK-801 from rat cortex homogenate. (TIF)

S3 Fig Competitive binding curves of the five target 1,2-diarylethylamines. (TIF)

S4 Fig GC/MS of 2-Cl-DPH. Top window shows the GC trace (retention time: 14.77 min) and bottom window shows the MS EI fragmentation of 2-Cl-DPH.
(TIF)

S5 Fig EI and CI ionization of 2-Cl-DPH. (TIF)

S6 Fig ^1^H NMR spectra (400 mHz) of 2-Cl-DPH freebase (20 mg/mL) in CDCl_3_. (TIF)

S7 Fig ^13^C PENDANT NMR spectra (100 mHz) of 2-Cl-DPH freebase (20 mg/mL) in CDCl_3_. (TIF)

S8 Fig 2-D HMQC NMR spectra of 2-Cl-DPH freebase (20 mg/mL) in CDCl_3_. (TIF)

S9 Fig 2-D HMBC NMR spectra of 2-Cl-DPH freebase (20 mg/mL) in CDCl_3_. (TIF)

S10 Fig 2-D COSY-45 NMR spectra of 2-Cl-DPH freebase (20 mg/mL) in CDCl_3_. (TIF)

S11 Fig DPH and 2-MXP test purchases. (TIF)

S12 Fig DPH powder, 2-MXP powder and 2-MXP “pellet” from test purchases. (From left to right)
(TIF)

S13 Fig 2-MXP “pellet” and packaging from test purchases. (TIF)

S1 File Compound CPM [^3^H]-MK-801 competitive binding data. (XLSX)

S2 File 2-MXP fEPSPs selectivity normalized data. (XLSX)

S3 File 10 uM D-AP5 & DPH method fEPSPs normalized data. (XLSX)

S4 File Drugs 1 and 10 uM fEPSPs normalized data. (XLSX)

S5 File Pre-pulse inhibition Startle Data. (XLSX)

S6 File Synthesis and analysis of 2-Cl-DPH. (PDF)

S1 Table Radioligand and concentrations used for the NIMH PDSP receptor binding screening assays. (DOCX)
