# Supplementary figures and images for: Pharmacological Investigations of the Dissociative ‘Legal Highs’ Diphenidine, Methoxphenidine and Analogues
Source: PLoS One. 2016 Jun 17;11(6):e0157021. doi: 10.1371/journal.pone.0157021 (PMC4912077; doi:10.1371/journal.pone.0157021)

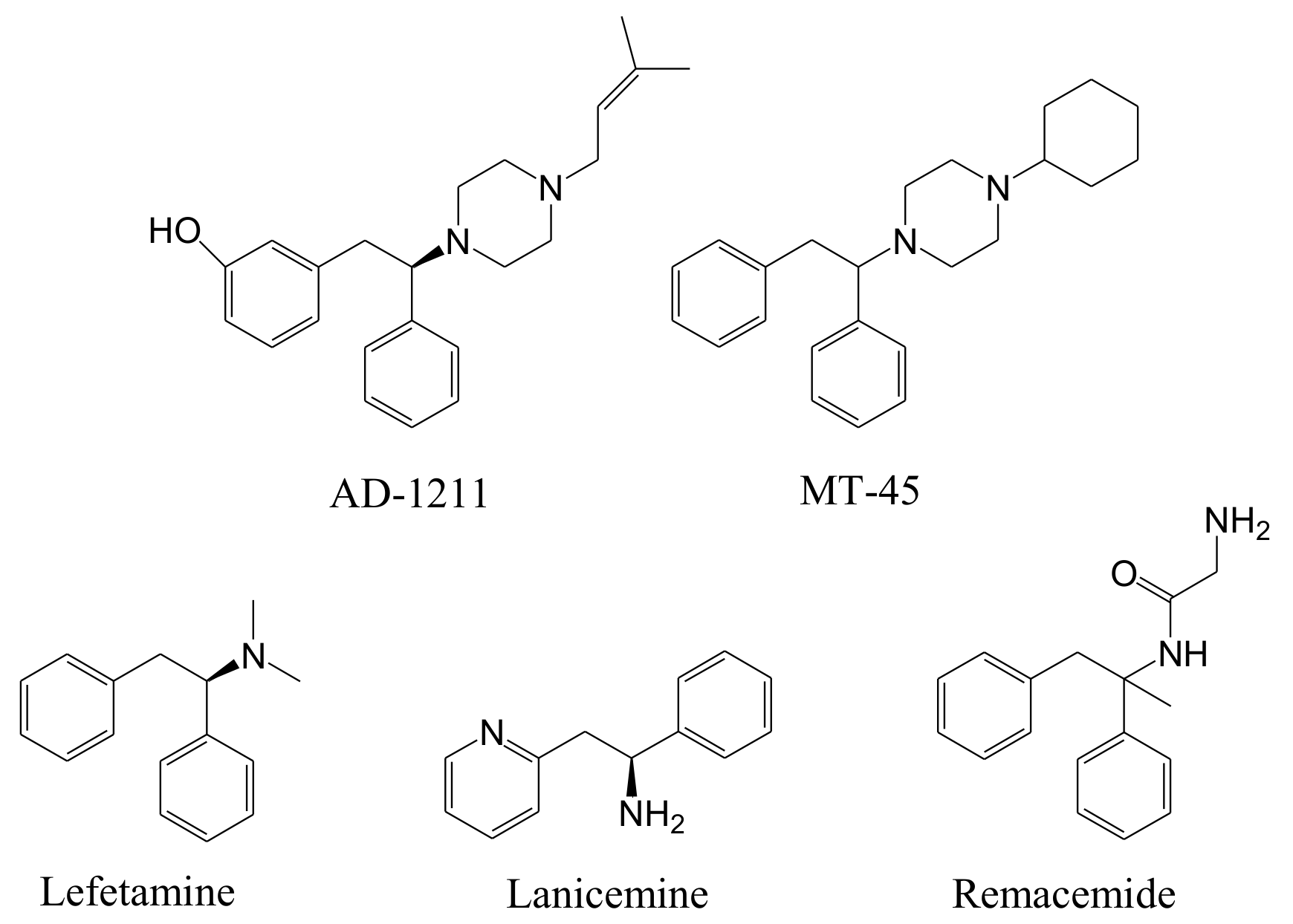

Supplement: S1 Fig — (TIF) [file pone.0157021.s001.tif]

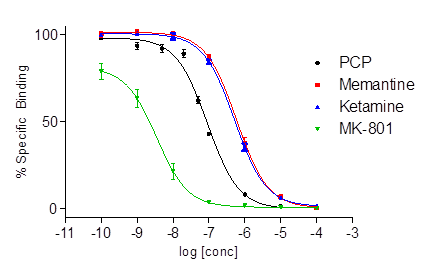

Supplement: S2 Fig — (TIF) [file pone.0157021.s002.tif]

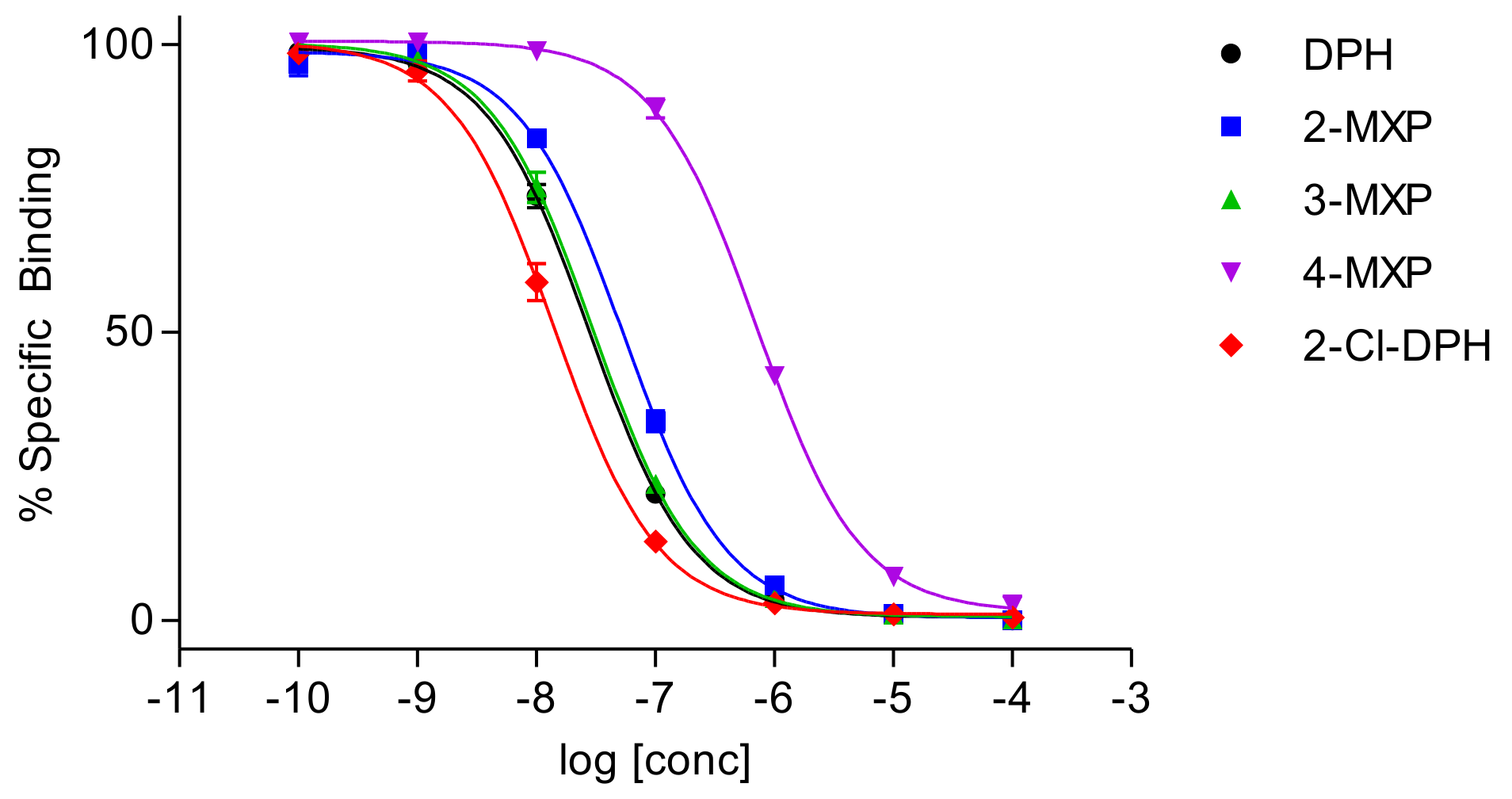

Supplement: S3 Fig — (TIF) [file pone.0157021.s003.tif]

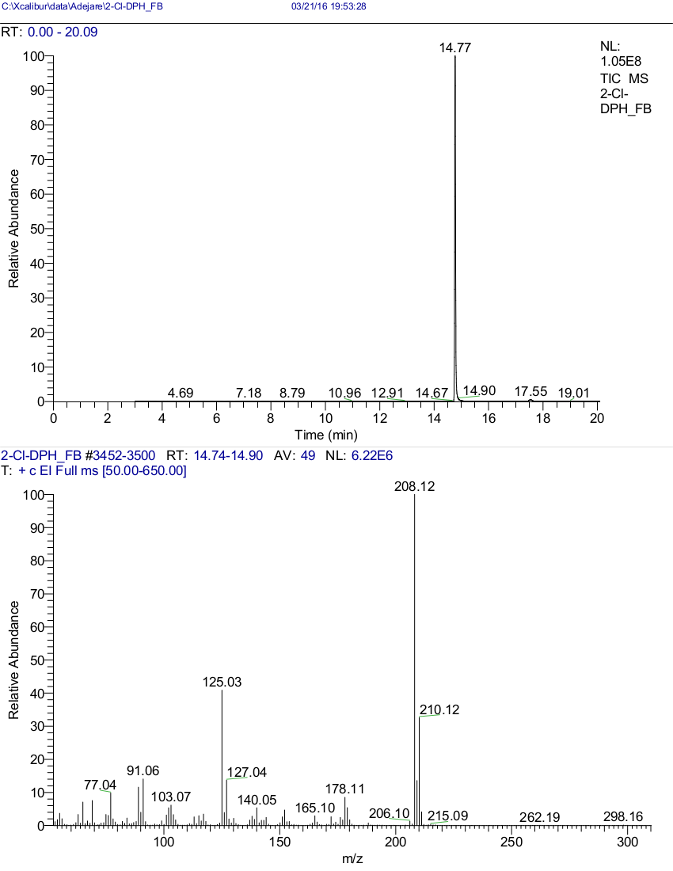

Supplement: S4 Fig — Top window shows the GC trace (retention time: 14.77 min) and bottom window shows the MS EI fragmentation of 2-Cl-DPH. (TIF) [file pone.0157021.s004.tif]

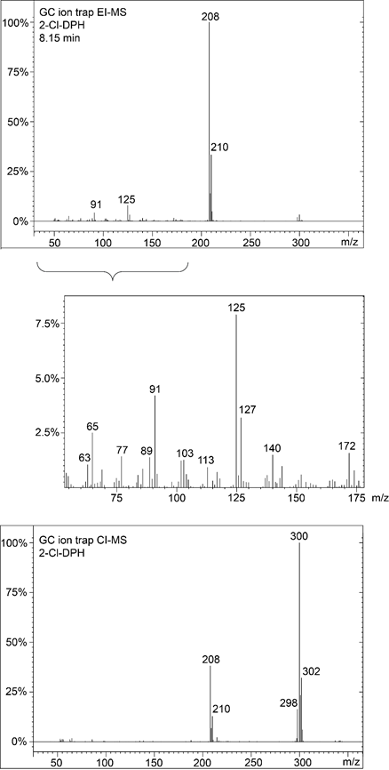

Supplement: S5 Fig — (TIF) [file pone.0157021.s005.tif]

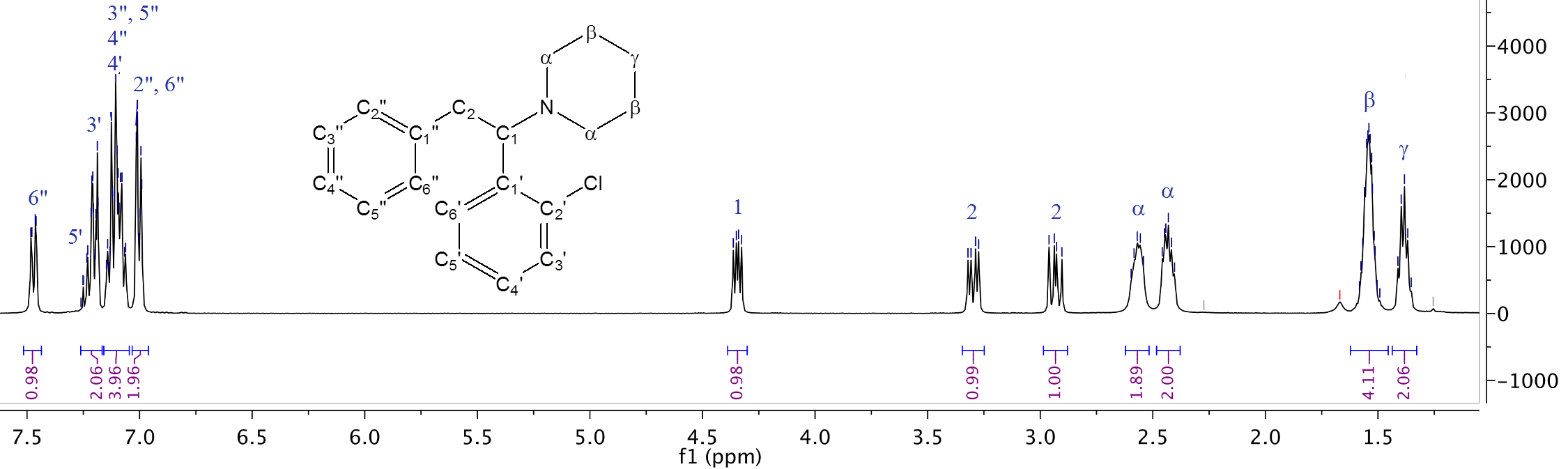

Supplement: S6 Fig — (TIF) [file pone.0157021.s006.tif]

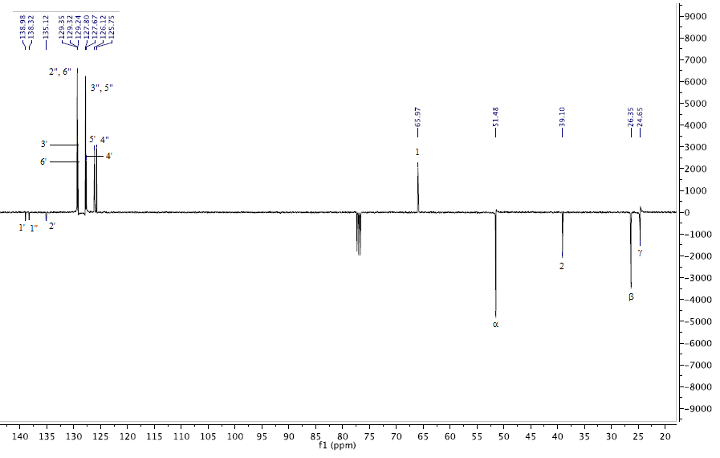

Supplement: S7 Fig — (TIF) [file pone.0157021.s007.tif]

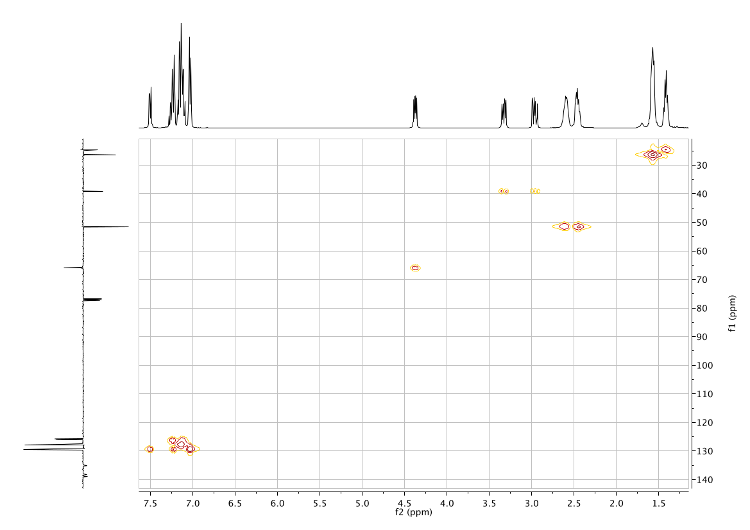

Supplement: S8 Fig — (TIF) [file pone.0157021.s008.tif]

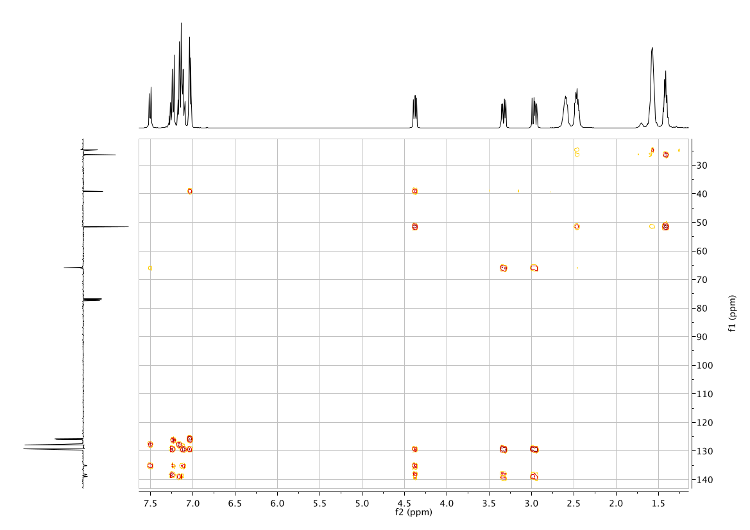

Supplement: S9 Fig — (TIF) [file pone.0157021.s009.tif]

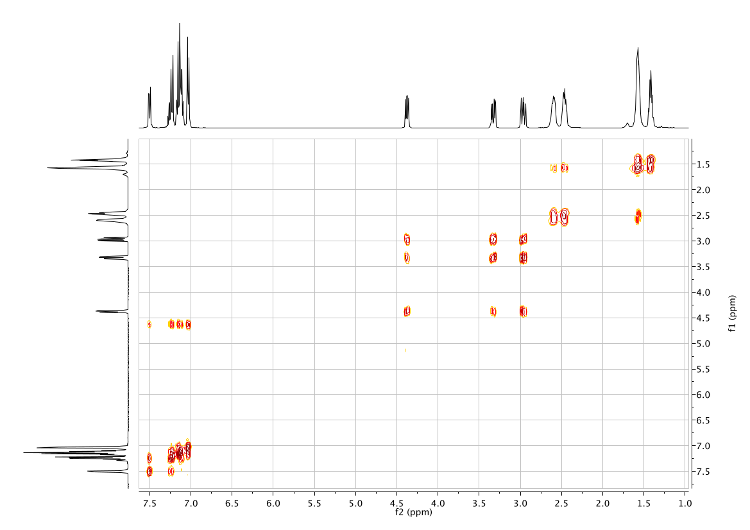

Supplement: S10 Fig — (TIF) [file pone.0157021.s010.tif]

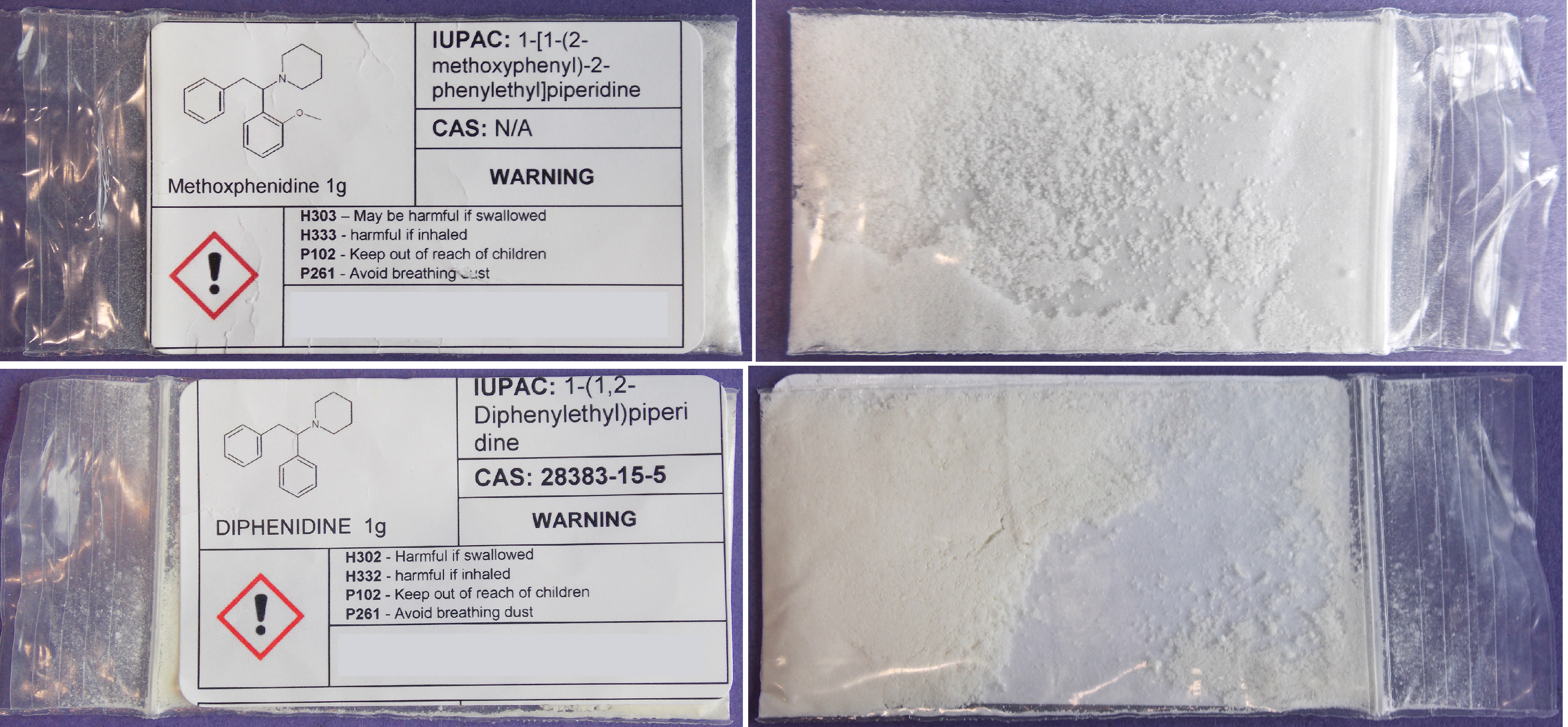

Supplement: S11 Fig — (TIF) [file pone.0157021.s011.tif]

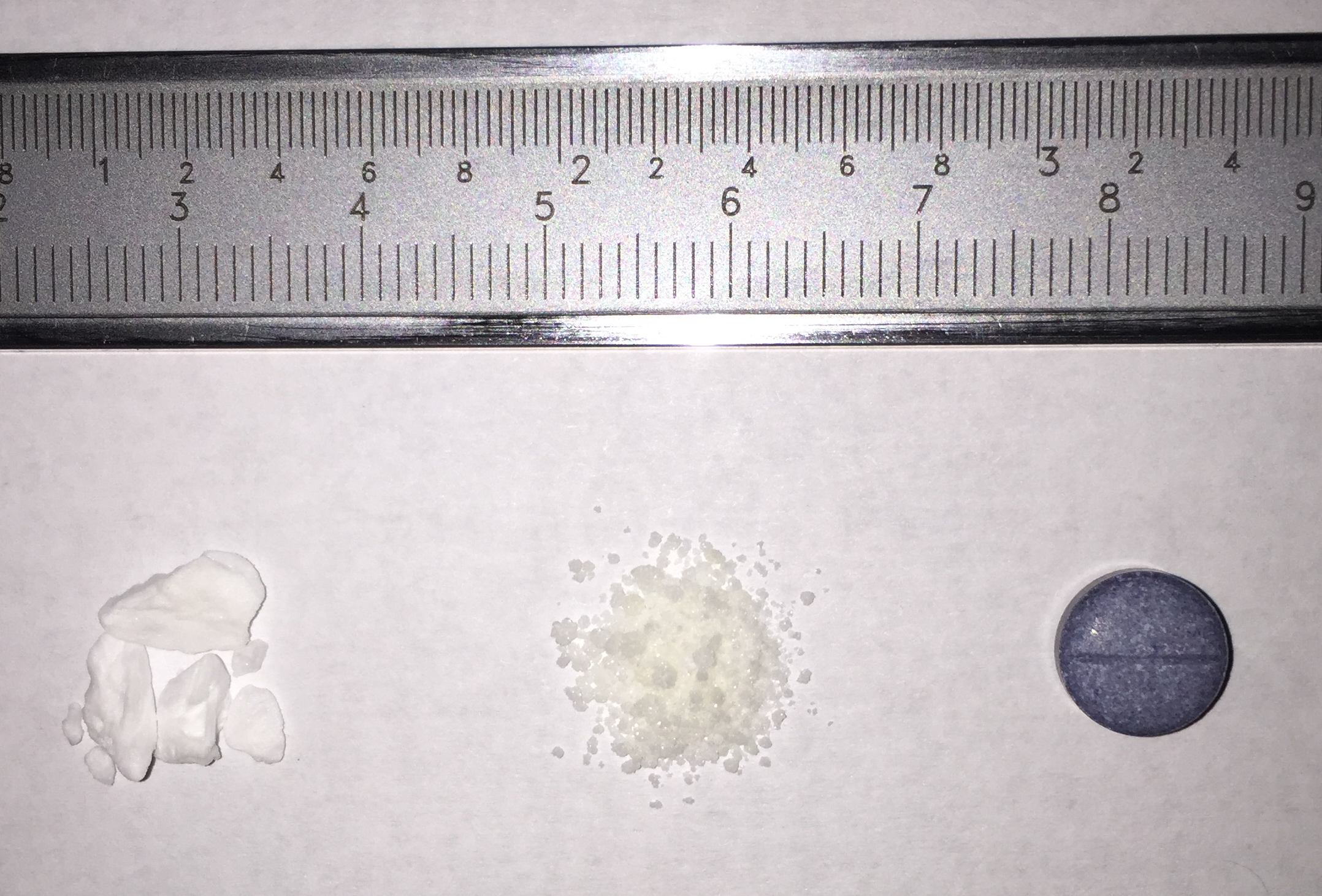

Supplement: S12 Fig — (From left to right) (TIF) [file pone.0157021.s012.tif]

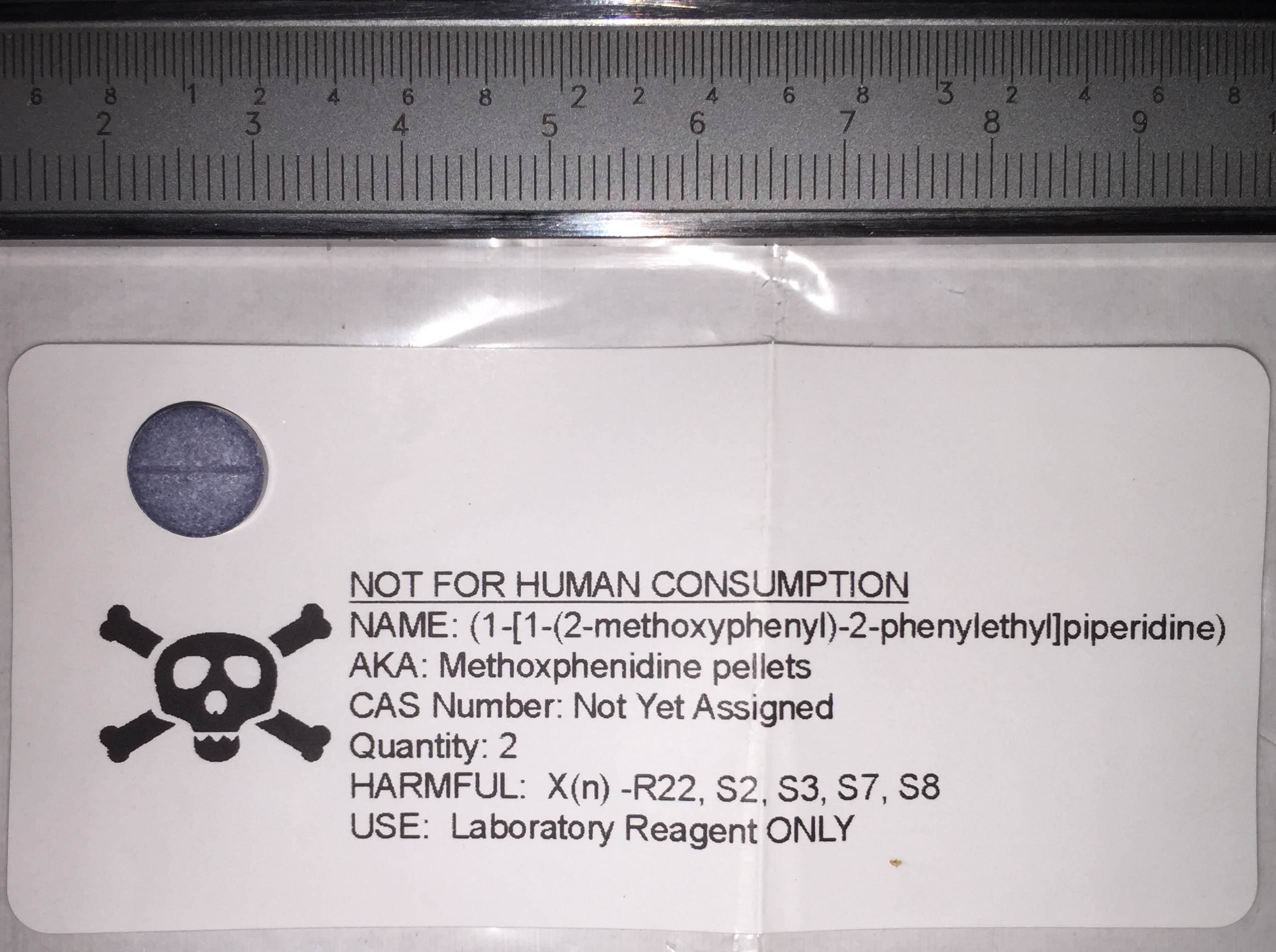

Supplement: S13 Fig — (TIF) [file pone.0157021.s013.tif]
