# Supplementary material for: Pharmacological Investigations of the Dissociative ‘Legal Highs’ Diphenidine, Methoxphenidine and Analogues
Source: PLoS One. 2016 Jun 17;11(6):e0157021. doi: 10.1371/journal.pone.0157021 (PMC4912077; doi:10.1371/journal.pone.0157021)
Supplement: S6 File — (PDF) [file pone.0157021.s019.pdf]

Synthesis of 2-Cl-diphenidine (2-Cl-DPH)

Materials

All starting materials, reagents and solvents for synthesis (≥ 95%) were obtained from Sigma-Aldrich (St. Louis, USA) except for Zinc dust which was obtained from Spectrum (Gardena CA, USA). Column chromatography was conducted using Merck silica gel, grade 9385 (230-400 mesh, 60 Å). Melting point ranges were obtained with a DigiMelt A160 SRS melting point apparatus (Stanford Research Systems, Sunnyvale, USA) at a ramp rate of 2 °C/min and are uncorrected.

Synthesis

To a solution of 40 mL dry acetonitrile (4 Å Molecular sieves) in a 100 mL round bottom flask under argon was added 1.96 g (30 mmol) zinc dust (325 mesh), 0.4 mL benzylbromide and 0.2 mL trifluoracetic acid and stirred for 5 min at room temperature (rt). 3 mL (25.3 mmol) benzylbromide, 0.988 mL (10 mmol) piperidine and 1.24 mL (11 mmol) 2-chlorobenzaldehyde were then added in rapid succession. The reaction was stirred at ambient temperature for 1 h at which point TLC and MS showed the reaction was complete. The reaction was quenched by pouring into 150 mL of a 5% NaOH solution and extracted with dichloromethane (2 x 100 mL). The combined organic extractions were dried with Na_2SO_4 and evaporated under vacuum to give a yellow oil. This oil was dissolved in 150 mL Et_2O and 0.75 mL 95% H_2SO_4 was added drop wise. After 5 min the sulfate salt precipitated out and was collected by vacuum filtration, washed with Et_2O and dried. The salt was recrystallized once by dissolving in a minimum amount of MeOH and diluting with 10 volumes of Et_2O. The crystals were collected by vacuum filtration and dried. The sulfate salt was converted to the freebase by dissolving in 200 mL H_2O. Once dissolved the solution was made basic to pH >12 with a concentrated KOH solution and extracted with ethyl acetate (3 x 100 mL). The organic extractions were pooled, washed with saline, dried with Na_2SO4 and evaporated under vacuum to give a colorless oil. (The sulfate salt step could likely be skipped and the crude freebase purified directly with column chromatography). The freebase was purified using silica gel flash column chromatography with a mobile phase of 4:1 (hexanes:ethyl acetate) containing 0.2% triethylamine. The desired fractions (TLC and MS) were pooled and evaporated to give 1.8 g of a colorless oil (56.3 % yield).

HCl salt: The freebase was dissolved in 20 mL ethanol (200 proof) and titrated to ~pH = 1 with concentrated HCl. The solvents were evaporated under warm air. Additional ethanol was added and evaporation repeated until free of moisture and excess acid. The resulting white solids were washed with ethyl acetate (2 x 5 mL) and dried with gentle heat. The solids were then recrystallized by dissolving in warm ethanol (10 mL) and diluting with Et_2O (20 mL) and storing at 0 °C. Crystals were collected the next day by decanting the solvent, washed with 5 mL ethyl acetate and dried in the oven at 60 °C. This was repeated twice (3X total) to give white crystalline solids. mp: 200.8-201.5 °C.

^{1}H NMR (400 mHz, $CDCl_3$): 7.47 dd(*J* = 7.7, 1.7 Hz, 6', 1-ArH), 7.20 m(5', H3'), 7.10 m(3,5'', 4'', 4', 3-ArH), 7.00 m(2'',6'', 2-ArH), 4.34 dd(*J* = 9.2, 5.5 Hz, 1, 1H), 3.30 dd(*J* = 13.5, 5.5 Hz, 2, 1H), 2.93 dd(13.5, 9.3 Hz, 2, 1H), 2.62-2.52 m(α, 2H), 2.48-2.38 m(α, 2H), 1.62-1.45 m(β, 4H), 1.38 p(*J* = 6.0 Hz, ɣ, 2H).

^{13}C NMR (100 mHz, $CDCl_3$): 138.98 (1'', 1-ArC), 138.32 (1', 1-ArC), 135.12 (2', 1-ArC), 129.35 (2'', 6'', 2-ArC), 129.32 (3'), 129.24 (6', 1-ArC), 127.80 (3'', 5'', 2-ArC), 127.67 (4', 1-C), 126.12 (5', 1-C), 125.75 (4'', 1-C), 65.97 (1, 1-C), 51.48 (α, 2-C), 39.10 (2, 1-C), 26.35 (β, 2-C), 24.65 (ɣ, 1-C).

Instrumentation

Nuclear magnetic resonance spectroscopy (S6, S7, S8, S9, S10 Fig.)

^{1}H (400 MHz) and ^{13}C NMR spectra (100 MHz) were obtained from 2-Cl-DPH freebase in $CDCl_3$ solution (100% and 99.96% D, 0.03% (v/v) TMS) at a concentration of 20 mg/mL. Spectra were recorded on a Bruker Ultrashield 400 plus spectrometer with a 5 mm BBO S1 (Z gradient plus) probe at 24 °C. Internal chemical shift references were TMS (δ = 0.00 ppm) and solvent (δ = 77.0 ppm).

Gas chromatography ion trap mass spectrometry (S2 Fig.)

Spectra (1.0 mg/mL in methanol) were recorded in electron ionization (EI) mode. A Thermo Trace 1300 GC gas chromatograph coupled to a Thermo ISQ_{QD} single quad mass spectrometer was employed in split mode (1:100) (ThermoFisher Scientific, USA). Thermo Xcalibur software, version 3.1, was used for data acquisition. Transfer line and ion source temperatures were set at 210 and 200 ºC, respectively. The carrier gas was helium at a flow rate of 1 mL/min. A Thermo TG-SQC column (15 m x 0.25mm x 0.25μm) was used for separation. The starting temperature was set at 100 ºC and was increased 8 ºC/min to 220 ºC then held constant for an additional 4 min to give a total run time of 20 min.

Gas chromatography ion trap mass spectrometry (S3 Fig.)

Spectra (0.5 mg/mL in methanol) were recorded in EI and chemical ionization (CI) mode using HPLC grade methanol as the liquid CI reagent (scan range m/z 41 – m/z 500). A Varian 450-GC gas chromatograph coupled to a Varian 220-MS ion trap mass spectrometer and a Varian 8400 autosampler was employed with a Varian CP-1177 injector (275 ºC) in split mode (1:50) (Walnut Creek, CA, USA). The Varian MS Data Review function of the Workstation software, version 6.91, was used for data acquisition. Transfer line, manifold and ion trap temperatures were set at 310, 80 and 220 ºC, respectively. The carrier gas was helium at a flow rate of 1 mL/min using the EFC constant flow mode. The default settings for CI ionization parameters (0.4 s/scan) were used: CI storage level *m/z* 19.0; ejection amplitude *m/z* 15.0; background mass *m/z* 55; maximum ionization time 2000 µs; maximum reaction time 40 ms; target TIC 5000 counts. An Agilent J&W VF-5ms GC column (30 m × 0.25 mm, 0.25 µm) was employed for separation. The starting temperature was set at 130 ºC and held for 1 min. The temperature then increased at 20 ºC/min to 280 ºC and held constant for 11.50 min to give a total run time of 20.00 min.
