# Supplementary material for: Pharmacological Investigations of the Dissociative ‘Legal Highs’ Diphenidine, Methoxphenidine and Analogues
Source: PLoS One. 2016 Jun 17;11(6):e0157021. doi: 10.1371/journal.pone.0157021 (PMC4912077; doi:10.1371/journal.pone.0157021)
Supplement: S1 Table — (DOCX) [file pone.0157021.s020.docx]

| Receptor | Radioligand  (concentration) | Receptor | Radioligand  (concentration) |
| --- | --- | --- | --- |
| 5-HT1A | [^3^H]8-OH-DPAT (0.5 nM) | H3 | [^3^H]-alpha-methylhistamine (0.4 nM) |
| 5-HT1B | [^3^H]GR127543 (0.3 nM) | H4 | [^3^H]Histamine (5 nM) |
| 5-HT1D | [^3^H]GR127543 (0.3 nM) | SERT | [^3^H]Citalopram (0.5 nM) |
| 5-HT1E | [^3^H]5-HT (3 nM) | NET | [^3^H]Nisoxetine (0.5 nM) |
| 5-HT2A | [^3^H]Ketanserin (0.5 nM) | DAT | [^3^H]WIN35428 (0.5 nM) |
| 5-HT2B | [^3^H]LSD (1 nM) | BZP | [^3^H]Flunitrazepam (0.5 nM) |
| 5-HT2C | [^3^H]Mesulergine (0.5 nM) | Alpha1A | [^3^H]Prazosin (0.7 nM) |
| 5-HT3 | [^3^H]LY278584 (0.3 nM) | Alpha1B | [^3^H]Prazosin (0.7 nM) |
| 5-HT5a | [^3^H]LSD (1 nM) | Alpha2A | [^3^H]Clonidine (1 nM) |
| 5-HT6 | [^3^H]LSD (1 nM) | Alpha2B | [^3^H]Clonidine (1 nM) |
| 5-HT7 | [^3^H]LSD (1 nM) | Alpha2C | [^3^H]Clonidine (1 nM) |
| D1 | [^3^H]SCH233930 (0.2 nM) | Beta1 | [^125^I]Iodopindolol (0.1 nM) |
| D2 | [^3^H]N-methylspiperone (0.2 nM) | Beta2 | [^125^I]Iodopindolol (0.1 nM) |
| D3 | [^3^H]N-methylspiperone (0.2 nM) | Beta3 | [^125^I]Iodopindolol (0.1 nM) |
| D4 | [^3^H]N-methylspiperone (0.2 nM) | M1 | [^3^H]QNB (0.5 nM) |
| D5 | [^3^H]SCH233930 (0.2 nM) | M2 | [^3^H]QNB (0.5 nM) |
| DOR | [^3^H]DADLE (0.3 nM) | M3 | [^3^H]QNB (0.5 nM) |
| KOR | [^3^H]U69593 (0.3 nM) | M4 | [^3^H]QNB (0.5 nM) |
| MOR | [^3^H]DAMGO (0.3 nM) | M5 | [^3^H]QNB (0.5 nM) |
| H1 | [^3^H]Pyrilamine (0.9 nM) | Sigma-1 | [^3^H]Pentazocine (3 nM) |
| H2 | [^3^H]Tiotidine (3 nM) | Sigma-2 | [^3^H]DTG (3 nM) |
| PBR | [^3^H]PK11195 (1 nM) |  |  |

Additional experimental details available in NIMH PDSP protocol book. [1]
